# Anticoagulated patient’s perception of their illness, their beliefs about the anticoagulant therapy prescribed and the relationship with adherence: impact of novel oral anticoagulant therapy – study protocol for The Switching Study: a prospective cohort study

**DOI:** 10.1186/s12878-016-0061-9

**Published:** 2016-08-23

**Authors:** Vivian Auyeung, Jignesh P. Patel, John K. Abdou, Bipin Vadher, Lynda Bonner, Alison Brown, Lara N. Roberts, Raj K. Patel, Roopen Arya

**Affiliations:** 1 King’s Thrombosis Centre, Department of Haematological Medicine, King’s College Hospital NHS Foundation Trust, London, UK; 2 Institute of Pharmaceutical Science, Faculty of Life Sciences & Medicine, King’s College London, 5th Floor, Franklin-Wilkins Building, London, SE1 9NH UK

**Keywords:** Anticoagulants, Atrial fibrillation, Beliefs, Illness perceptions, Medication adherence, Venous thromboembolism, Quality of life

## Abstract

**Background:**

Anticoagulant therapy is prescribed for millions of patients worldwide for the prevention and treatment of both arterial and venous thrombosis. Historically, only vitamin K antagonists have been available for clinicians to prescribe. The anticoagulation landscape is changing. The recent availability of the novel oral anticoagulants overcome many of the disadvantages associated with vitamin K antagonists. However the lack of formal monitoring and clinic follow-up is a concern for clinicians, as medication adherence is being assumed, which is known to decline in patients prescribed medications for chronic conditions. The switching study is a programme of work investigating the association between medication adherence and patient’s beliefs about anticoagulation therapy (warfarin and subsequently novel oral anticoagulants), together with beliefs about their illness and anticoagulation related quality of life.

**Methods/design:**

The anticoagulation database at King’s College Hospital will be interrogated and two groups of patients will be identified; those with a time in therapeutic range on warfarin of ≥75 % and those <50 %. These groups of patients will have their illness perceptions, anticoagulation specific quality of life and beliefs about medications compared. Those patients in the time in therapeutic range <50 % group, will be then be invited to switch to a novel oral anticoagulant, as per local guidance. Those patients, who do switch, will then be followed longitudinally and have their adherence, illness perceptions, anticoagulation specific quality of life and beliefs about medications, re-evaluated on the novel agent. The results from these sub-studies, will inform a clinical pathway to support patients on these novel agents, which will be evaluated in an independent group of patients.

**Discussion:**

The results from the switching study will be used to develop a clinical pathway to support patient’s prescribed novel oral anticoagulant therapy long-term.

**Electronic supplementary material:**

The online version of this article (doi:10.1186/s12878-016-0061-9) contains supplementary material, which is available to authorized users.

## Background

Anticoagulant therapy is prescribed for millions of patient’s world-wide, most commonly for the acute treatment and long-term prevention of venous thromboembolism (VTE) and as primary and secondary prevention of stroke in the context of atrial fibrillation (AF) [1, 2]. Until recently the majority of patients requiring chronic anticoagulant therapy were prescribed vitamin K antagonists (VKA), as these were the only oral anticoagulant agents available [1, 2]. VKA therapy comes with some practical disadvantages; need for monitoring and a relatively significant number of drug-drug and drug-food interactions, which has meant that not all patients eligible have benefitted from these agents in the past [3]. In recent years, the aforementioned disadvantages has led to the development of new classes of oral anticoagulants, the direct thrombin inhibitor (dabigatran) and the direct Xa inhibitors (apixaban, betrixaban, edoxaban, rivaroxaban). These agents hold many advantages over VKA, primarily of a predictable pharmacokinetic nature, meaning that there is minimal requirement for regular monitoring of anticoagulant effect [4]. In the United Kingdom (UK), the available direct oral anticoagulants, apixaban, dabigatran, edoxaban and rivaroxaban are all approved for AF and VTE indications and available for clinicians to prescribe as treatment options, when clinically indicated [5–13]. Indeed their availability has sparked local initiatives to ensure that patients in AF with a CHA_2_DS_2_VASc score [14] of two or above are offered oral anticoagulation, be it with VKA or the novel agents, referred to herein as NOAC. The general perception is, that the NOACs are *easier* to use, so uptake both from a clinician (prescribing) and patient (receiving) perspective is likely to be higher, with cost analyses modelling suggesting that these agents are cost-effective [5–13]. Treatment pathways across the UK are being revisited to accommodate these agents and the advantages they bring. However, these agents are not free from risks. Compared to VKA, little experience exists on how these agents perform long-term, e.g. in minimising complications associated with VTE such as the post-thrombotic syndrome. Additionally, there are currently no antidotes available for the direct Xa inhibitors; a situation which is likely to change in the coming months. Finally, the benefits cited for the NOACs assume that patients actually take the medication as prescribed. Research suggests that approximately 30–50 % of medication prescribed for chronic conditions are not taken as intended [15]; adherence is often found to be high during the initial months of therapy and then found to decline in many patients [16], with clinician’s ability to recognise medication non-adherence reported to be poor [17–20]. Medication non-adherence not only impacts negatively on the patient concerned, the wider health-care economy is adversely affected [21]. Research confirms that individual patient’s beliefs about medicines are a strong predictor of their adherence to treatment. These beliefs can be grouped under two categories; (1) perceptions of necessity and (2) concerns about negative effects [22]. Therefore beliefs that failure to take the treatment could result in adverse consequences is associated with higher adherence rates (and vice versa). Acknowledging that all patients will have differing beliefs about their medications is the first step in addressing the medication non-adherence problem [22, 23].

With the availability of NOAC therapy, an opportunity now exists to re-visit patients who are currently prescribed VKA with poor anticoagulation control, and consider switching their anticoagulant therapy to a NOAC. The question is how to determine *success* or *failure* on VKA. A common method of determining good anticoagulation control on VKA is through their time in therapeutic range (TTR). The Rosendaal method [24] is the most widely used method to calculate TTR and uses linear interpolation to calculate an INR value for each day between observed INR values (over a 1 year period). The TTR represents the percentage of these INR values in days that are in the therapeutic range. The TTR is a valid marker to use in clinic, as it has been reported to predict clinical outcomes such as major bleeding, stroke and systemic embolic events [25]. TTR provides a means, however imperfect [26], of identifying possible medication non-adherence and/or whether the patient may benefit from a switch to NOAC. In the UK, national guidelines suggest using the TTR criteria for determining which patients might be prioritised for switching to a NOAC. Indeed, the recently published National Institute for Care Excellence guidelines for AF stipulate that anticoagulation clinics should reassess anticoagulation for patients with poor anticoagulation control, and one of the criterion specified for re-assessment is if a patient’s TTR is <65 % [27].

Non-adherence in the context of anticoagulant therapy is not new and the issue has received attention from researchers in the past [28–32], with the importance of non-adherence to NOAC now emerging [33–35]. Little research exists on what patients general and specific concerns or necessity might be about the VKA that they are currently prescribed. This is important, as with a significant change in the anticoagulation landscape, understanding the patient perspective, particularly those with poor TTR, could help identify patterns of concerns that this group of patients may have, which could impact on their medication taking behaviour, if switched to a NOAC. Studying this would help with service delivery and determine if a specific clinical pathway would support adherence with chronic anticoagulant therapy in those identified or suspected of poor adherence - as currently, no long term pathway for NOAC patients is expected to be in place.

The Switching study is a series of prospective studies designed to investigate the association between medication adherence and patients’ beliefs about anticoagulation therapy (warfarin and subsequently NOAC) together with beliefs about their illness and anticoagulation-related quality of life.

The Switching study aims to test the following hypotheses:

- *Patients’ beliefs about warfarin and their illness perceptions is associated with their adherence to warfarin as measured by time in treatment range (TTR), over and above any clinical or demographic variables.*
- *Patients’ beliefs about NOAC at baseline and their illness perception can predict their adherence to NOAC, over and above any clinical or demographic variables.*
- *Patients with symptomatic disease (either VTE or AF) have higher necessity scores for anticoagulant treatment compared to patients with asymptomatic disease.*
- *Patients with a TTR >75 % have low concerns and higher necessity scores for warfarin than those with a TTR <50 %.*
- *Patient’s responses to the illness perception questionnaire (IPQ) is specific to treatment and will change, following a switch to NOAC.*
- *Patient’s quality of life scores improve following a switch to NOAC.*

## Methods/design

### Patients and patient selection

Anticoagulated patients who meet the inclusion criteria, defined by their TTR, will be identified through the DAWN® databases used at King’s College Hospital NHS Foundation Trust. King’s College Hospital is a large teaching hospital, based in South East London, providing specialist tertiary services of liver disease and transplantation, neurosciences, haemato-oncology, cardiology and foetal medicine. The anticoagulation service at King’s is provided between two hospital sites; Denmark Hill and Bromley. The combined anticoagulation population is ~6, 000 patients, with a clinic average TTR of 76 and 73 % for the Denmark Hill and Bromley sites respectively.

Patient selection for the studies will be dictated by their current time TTR, as per protocol and whether patients fit the eligibility criteria. The anticoagulant clinic at King’s utilises the DAWN® software, and the TTR is calculated for patient’s prescribed chronic anticoagulation, by reviewing INR values 1 year from the date of calculation. The TTR calculation utilises the Rosendaal method [24].

### Psychometric instruments

The instruments which will be administered to patients in this study comprise of the Beliefs about Medicines Questionnaire (BMQ) [36], the revised Illness Perception Questionnaire (IPQ-R) [37] and the Anti-Clot Treatment Scale (ACTS) [38] quality of life instrument. The BMQ is a validated instrument for assessing patient’s beliefs about medicines in general (overuse and harm) and their prescribed medication specifically (necessity and concerns). The IPQ-R is a validated instrument that assesses patient’s perception of their illness. The measures *identity -* the symptoms the patient associates with the illness, *cause* - personal ideas about aetiology, *time-line* - the perceived duration of the illness (chronicity) and how it fluctuates (cyclical), *consequences* - expected effects and outcome and *cure control* - how one controls or recovers from the illness (personal control) and the treatments available to manage it (treatment control). There is also a global measure of *illness coherence* – the degree to which the illness makes sense to the patient and *emotional representation* - a measure of the impact of the illness on their emotional well-being. The ACTS is a 17-item anticoagulation specific quality of life instrument which measures burden (a 12-item scale) and benefits (a 3 -item scale). The questionnaire packs administered to patients in the Switching study are available as Additional files 1, 2, 3 and 4 with this manuscript.

### Switching study series

#### Study I

For study I, two groups of patients prescribed chronic VKA for the primary and secondary prevention of stroke in the context of AF and for treatment and secondary prevention of VTE, will be recruited: those with a TTR >75 % (group 1) and those with a TTR <50 % (group 2). The TTR cut-off’s of >75 and <50 % were chosen to inform groups 1 and 2, based on the availability of the NOACs providing an opportunity to switch patients poorly controlled on VKA; it was felt that the patients with the poorest control should be given this option to switch first, i.e. be prioritised. Furthermore, having two distinct groups (<50 and >75 %) and understanding and appreciating the differences between these two groups will ensure that the hypotheses posed could be tested and if appropriate, a suitable support mechanism can be developed for patients prescribed chronic NOAC therapy where it is anticipated that adherence might be an issue.

For group 1 patients, their illness perception for why they are prescribed anticoagulant therapy, their beliefs about VKA and their anticoagulation specific quality of life explored through the aforementioned psychometric instruments [36–38], once through the post. Eligible patients will be consecutively identified from the DAWN® database. These patients will remain on VKA therapy, due to their excellent TTR.

In contrast, group 2 patients, due to their poor TTR will specifically be invited to clinic (visit 1) to have their beliefs about warfarin, their perception of their illness and their quality of life explored through the aforementioned validated instruments. Study I will assess the association between adherence to warfarin and i) patient beliefs about treatment and ii) patient perception of their illness and compare responses from group 1 and group 2 patients. In addition, a baseline level for patient’s anticoagulation-specific quality of life will be established.

#### Study II

Following completion of the psychometric instruments, patients in group 2 (from study I) will be asked if they wish to switch to a NOAC, according to local guidelines. Patients who then switch will prospectively be followed in clinic will be required to return for a clinic visit at 1, 2 months and 1 year into NOAC treatment allowing for the longitudinal assessment of adherence. At their 1-month follow-up visit (visit 2), patients will be given a questionnaire pack comprising of validated instruments designed to assess their newly established beliefs about NOAC therapy and any short-term changes in quality of life. The same instruments will be administered at their 1-year follow-up (visit 4) to determine whether any changes in beliefs, illness perceptions and quality of life are sustained. The questionnaire pack will be posted to the patient, prior to their clinic visit 4, requesting them to complete and bring along to their clinic appointment. To prevent participant burden, no questionnaires will be administered at the 2-month follow-up visit (visit 3). However, at 1-month and 2-month follow-up visits (visits 2 and 3), patients will be asked to bring in their NOAC medication to clinic, so a pill count can be conducted by the reviewing clinician.

Patients identified in this group who decline a switch to a novel agent, will also be followed longitudinally for the duration of the study and any clinically relevant outcomes will be reported. Furthermore, their reasons for not wishing to switch, will formally be recorded and described.

Analysis for studies I and II will be conducted on an intention to treat basis.

#### Study III

**Fig. 1:**
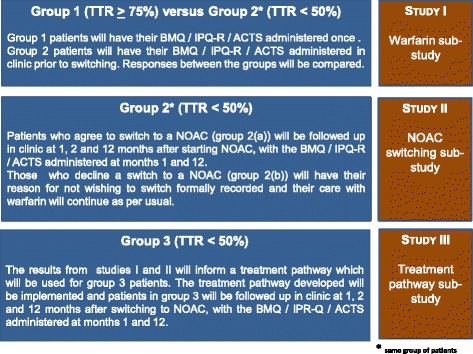
Summary of the three sub-studies which form the Switching Study programme

Following completion of studies I and II, the data collected will be analysed and used to design a clinical pathway which will be evaluated in a third (independent) group of patients (group 3 – patients with a TTR <50 %). The clinical pathway will be informed from the 2-month follow-up data collected up to visit 3 from patients in group 2 of study II. Group 3 patients will have the same follow-up in clinic as per the schedule outlined for group 2 patients, and analysis for study III will also be conducted on an intention to treat basis. The findings from group 2 patients (beliefs about medicines, illness perceptions, quality of life and adherence to anticoagulant therapy) will then be compared to the findings from group 3 patients. In this way, this controlled study design will then allow an assessment of the effectiveness of the clinical pathway developed. Figure 1 summarises the three studies which form this programme of work.

### Inclusion criteria

All patients with a TTR <50 % prescribed warfarin therapy (for recruitment to group 2 and group 3). Where the low TTR is not due recent hospital admissions, surgical procedures or significant drug changes; this will be determined, following initial identification of patients in this group.

A control group will also be recruited comprising of patients with a TTR >75 % (for recruitment to group 1).

### Exclusion criteria

Patients with a TTR >50–<75 % (for recruitment to group 2 and group 3).

Patients with a contra-indication to NOAC (i.e. abnormal liver function tests, CrCl <15 ml/min, taking concurrent interacting drugs which are contra-indicated).

Those patients unable to attend clinic for a formal consultation.

Those patients unable to read English.

Those patients who do not wish to participate.

Pregnant patients.

### Study outcomes

- Individual % medication adherence to direct acting anticoagulant therapy (1 year following switch)
- Changes in patients beliefs about the anticoagulation therapy prescribed, at 1 month and 1 year following a switch to NOAC therapy
- Changes in patients illness perceptions (1 month and 1 year following switch)
- Changes in patients anticoagulation specific quality of life (1 month and 1 year following switch)
- Persistence with NOAC therapy, following a switch from VKA (1 year following switch)

### Data collection

Table 1 outlines the study visit schedule and what instruments will be administered when for study II, including details of clinical information collected for patients who consent to the study. For study III, a new group of patients will be recruited, with the study visit schedule also following the schedule outlined in Table 1.

**Table 1:** Schedule of visits for patients recruited to studies II and III

*Visit 1 (baseline – on VKA)*	*Visit 2 (1 month into NOAC)*	*Visit 3 (2 months into NOAC)*	*Visit 4 (1 year into NOAC)*
Full medical and drug history obtained, including calculation of ^a^CHADS_2_ [44], ^a^CHA_2_DS_2_VASc [14], HASBLED [45], the Medication Related Complexity Index (MRCI) [46], SAMe-TT_2_R_2_ [26], the Charlson index [47] and whether the patient has symptomatic disease or not	Patient reviewed clinically	Patient reviewed clinically	IPQ/BMQ (NOAC)/ACTS instruments administered
Socio-demographic information^b^	Patient self-reported missed doses recorded + pill count	Patient self-reported missed doses recorded + pill count	PTS assessment if DVT patient
Informed written consent	Another 1 month prescription of NOAC given to patient	Another 1 month prescription of NOAC given to patient	Adherence assessment through GP summary care record, patient self-report and the 8-item Morisky Medication Adherence Scale
IPQ/BMQ (warfarin)/ACTS instruments administered	Bloods for UEs/FBC	Bloods for UEs/FBC (if not done at visit 2)	
Patient switched to NOAC (1 month prescription)	IPQ/BMQ (NOAC)/ACTS instruments administered	Patient transferred to GP for on-going prescriptions	
PTS assessment if DVT patient	PTS assessment if DVT patient	PTS assessment if DVT patient	

^a^only for patients prescribed anticoagulant therapy in the context of atrial fibrillation

^b^socio-demographic information will be obtained from the patient’s medical notes, namely age, gender, post-code, ethnicity

For studies II and III, patients will be reviewed by pharmacists trained in the field of anticoagulation working at King’s College Hospital. The questionnaire will be administered by the pharmacist reviewing the patient in clinic, with patients offered the option of completing the questionnaire in clinic or taking home and returning at their subsequent clinic visit. Patients will be advised that the questionnaires will take approximately 20 min to complete.

Patients who are prescribed long-term anticoagulation for the secondary prevention of VTE, and whose primary VTE event was a deep vein thrombosis, will at each clinic visit, have an assessment of post-thrombotic syndrome (PTS) using the Villalta score [39], as part of their clinical review at clinic visits.

### Adherence to NOAC

During the first 2 months of therapy, adherence to NOAC will be recorded through a pill count at the 1 and 2 month follow-up visits (visits 2 and 3). This pill count will be conducted by the pharmacist reviewing the patient in clinic and will compare the number of tablets the patient has to the number of tablets the patient is expected to have, following the commencement of NOAC. When patients are reviewed in clinic at 1 year into their NOAC therapy, specific adherence screening questions will be asked to assess adherence during months 4 to 12 of NOAC therapy [40] in addition to asking patients how many doses of their anticoagulation they have missed during the last week. The adherence screening questions at this visit are informed from the 8-item Morisky Medication Adherence Scale [41]. Furthermore, in order to assess persistence with their anticoagulation therapy, patient’s summary care record and prescriptions records will be reviewed, allowing anticoagulation coverage to be calculated, during the period which the GP was prescribing the NOAC for the patient, in order to measure persistence up to this time point.

### Consent, confidentiality and data storage

Group 1 patients will be sent the study patient information leaflet, along with the study questionnaire and a pre-paid envelope, to return the completed questionnaire to the research team.

When group 2 patients are invited for the consultation in the anticoagulation clinic, they will be sent the study patient information and asked to read it prior to their clinic visit. At the clinic visit, the pharmacist in clinic will ask the patient if they wish to participate in the study and address any questions they may have. For those patients who indicate that they interested, the pharmacist will recruit them into the study. Informed written consent will be taken by the pharmacist. For those patients who do not wish to participate, the pharmacist will still offer the patient the opportunity to switch to a NOAC, as per local guidance.

A similar process to that of group 2 patients will be followed for group 3 patients, at the appropriate time for study III.

All recruited patients will be reviewed in Haematology outpatients at the respective hospitals, by pharmacists trained in the study.

Data collected for the purposes of the study will be recorded by the pharmacist on a pre-determined study form, which captures the information required for the study. At the earliest opportunity, the principal researcher associated with the study will transfer the data to an excel database at the respective hospitals and subsequently transfer to an SPSS database, with the hard copy of the data collection form being filed and stored in a locked office within the haematology department at King’s College Hospital, with only members of the clinical team having access to this office.

One week after entry of a patient’s data onto the SPSS database, the data which had been entered will be re-checked for any entry discrepancies by the principal researcher.

As soon as a patient consents to participate in the study, they will be given a unique study number. This number will be entered onto the SPSS database and will only be traceable back to an individual patient by accessing the study form for the patient in the haematology department at King’s College Hospital.

All electronic data will be encrypted and password protected.

Only direct members of the research team will have access to the full set of electronic data generated within the study. The completed questionnaires for studies I, II and III will be stored within a locked office within the haematology department at King’s College Hospital.

### Sample size and statistical analysis

#### Study I & II

Patients who are >75 % in TTR (group 1) will be classified as adherent and those who are <50 % in TTR (group 2) will be classified as non-adherent. To determine if patients’ beliefs about warfarin and their illness perception is associated with their adherence status, univariate analyses will first be conducted to identify the key variables that will be entered into the multiple logistic regression model. Assuming a recruitment ratio of 1:1 of non-adherent : adherent patients, a total sample size of 180 to 240 patients will be able to accommodate 6 to 8 predictive variables. i.e. up to 120 patients who are adherent and up to 120 patients who are non-adherent are required as measured by TTR.

Multiple linear regression will be used to examine whether illness and treatment beliefs can predict adherence to NOAC. Using the formula that 15 observations are needed per degree of freedom, a sample size of 240 will be able to test whether illness and treatment beliefs (11 dimensions) and up to five demographic/clinical variables can predict adherence to NOAC. Therefore a sample size of 240 patients who are switched to NOAC is required.

However, as the refusal rate for switching to NOAC for eligible patients is not known, and given that the NOAC patient sample will derive from those who are non-adherent to warfarin (<50 % TTR), at least 240 patients – rather than 120 patients - who are non-adherent will need to be recruited in order to be able to perform both the linear regression analysis and logistic regression analysis. A conservative refusal rate of 30 % would suggest 342 patients who are <50 % TTR on warfarin would need to be recruited into group 2.

#### Study III

Three hundred forty-two patients will be recruited into group 2 which will act as the control group. Power calculations (GPower 3.1) assuming one-tailed analysis using an independent groups design, suggest that a 1:1 matching i.e. recruiting 342 patients into the intervention group (group 3) would allow for the detection of a small-sized effect of the intervention (d = 0.20).

The Switching study has been reviewed and given a favorable opinion by the London-Dulwich Research Ethics Committee: REC reference: 13/LO/1468.

## Discussion

The Switching study aims to understand patient’s perception of their illness, their beliefs about medication and their anticoagulation specific quality of life, as it relates to adherence to anticoagulant therapy. We are not the first to study this in a population anticoagulated with VKA. Of particular note is the recently published TREAT study [42, 43]. This study was a randomised controlled trial of an intensive educational intervention for patients requiring VKA for stroke prevention in the context of AF. The study randomised warfarin-naïve patients to usual care and the intervention or usual care alone. The study administered the BMQ and IPQ-R [36, 37] to patients at baseline, 1, 2, 6 and 12 months post intervention, and found that a theory based educational intervention significantly improves TTR in patients during the first 6 months, although these benefits did not appear to be maintained longer-term. This study demonstrates the utility of validated psychometric instruments in understanding and designing interventions that improve anticoagulation control in patients, however the results also demonstrate that more work is needed to understand techniques that can support patients in maintaining effective levels of self-management, particularly with the use of NOAC therapy.

The availability of NOACs, has led to a drive in the UK to ensure that patients who would benefit from oral anticoagulation, are identified and offered appropriate treatment; this is particularly the case for stroke prevention in the context of AF. However, although these agents are *easier* to use, both from a clinician and patient perspective, the lack of formal clinic follow-up and re-enforcement of adherence could be a problem, given what is already known about non-adherence to medication for chronic conditions. Current cost-analysis which state that these NOAC are cost-effective [5–13], assume full adherence to medication. This is clearly not a true reflection of clinical practice. Therefore understanding patients views about their illness, their beliefs about the anticoagulant prescribed and their anticoagulation-related quality of life, should help us to understand what type of support patients prescribed both VKAs and NOAC long-term might require to support adherence. This should not only benefit the patient, it should help ensure commissioners obtain value for money, given the current and likely future financial constraints within the health service.

Our study began recruitment in July 2014 and the full results are expected in 3 years.

## Abbreviations

ACTS, anti-clot treatment scale; BMQ, beliefs about medication questionnaire; CHA_2_DS_2_VASc, congestive heart failure, hypertension, age ≥75 years (2), stroke (2), vascular disease, age 65–74 years, sex category (female (1)); CHADS_2_, congestive heart failure, hypertension, age ≥75 years, stroke (2); CrCl, creatinine clearance; DVT, deep vein thrombosis; FBC, full blood count; GP, general practitioner; HASBLED, hypertension, abnormal renal or liver function, stroke, bleeding, labile INRs, elderly, drugs or alcohol; INR, International Normalised Ratio; IPQ, illness perceptions questionnaire; MRCI, medication related complexity index; NOAC, novel oral anticoagulant; PTS, post-thrombotic syndrome; SAMe-TT_2_R_2_, sex (female), age (<60 years), medical history, treatment (interacting drugs), T (tobacco use within 2 years (2)), race (non-white (2)); TTR, time in therapeutic range; UEs, urea and electrolytes; VKA, Vitamin K antagonists

## Additional files

Additional file 1: Questionnaire packs administered to patients prescribed warfarin. (PDF 276 kb) Additional file 2: Questionnaire packs administered to patients prescribed apixaban. (PDF 276 kb) Additional file 3: Questionnaire packs administered to patients prescribed dabigatran. (PDF 276 kb) Additional file 4: Questionnaire packs administered to patients prescribed rivaroxaban*. (PDF 277 kb)
