# Supplementary material for: Anticoagulated patient’s perception of their illness, their beliefs about the anticoagulant therapy prescribed and the relationship with adherence: impact of novel oral anticoagulant therapy – study protocol for The Switching Study: a prospective cohort study
Source: BMC Hematol. 2016 Aug 23;16(1):22. doi: 10.1186/s12878-016-0061-9 (PMC4995624; doi:10.1186/s12878-016-0061-9)
Supplement: Additional file 2: — Questionnaire packs administered to patients prescribed apixaban. (PDF 276 kb) [file 12878_2016_61_MOESM2_ESM.pdf]

Your views on the apixaban therapy prescribed

You have been sent this questionnaire, as you are currently prescribed apixaban therapy. Currently, very little information exists about patient's views on having to take apixaban.

As part of a research study we are conducting within the anticoagulant clinic at King's College hospital, we want to explore your views about apixaban, and would like you to complete this questionnaire.

The questionnaire comprises of three main sections. At the beginning of each section, a short explanation will precede the questions and provide specific instructions on how to complete.

Please answer every question. It will take approximately 20 minutes to complete.

Your answers will be completely anonymous and will be kept confidential.
There are no "right" answers to the questions – we are simply interested in your views.

Thank you for taking the time to complete this questionnaire

Section 1:

Questions relating to medicine use in general

This section explores your views and concerns (if any) about taking medicines in general.

Please answer every question by ticking the box that best describes your views to each statement.

Please remember, there are no “right” answers to the questions, we are simply interested in your views.

		Strongly agree	Agree	Uncertain	Disagree	Strongly disagree
G1	Doctors use too many medicines					
G2	Patients who take medicines should stop their treatment for a while every now and again					
G3	Most medicines are addictive					
G4	Natural remedies are safer than medicines					
G5	Medicines do more harm than good					
G6	All medicines are poisons					
G7	Doctors place too much trust on medicines					
G8	If doctors had more time with patients they would prescribe fewer medicines					

Section 1 continued:

Specific questions relating to apixaban

The following questions explore your views and concerns (if any) around taking apixaban specifically.

Please answer every question by ticking the box that best describes your views to each statement.

Please remember, there are no “right” answers to the questions, we are simply interested in your views.

		Strongly agree	Agree	Uncertain	Disagree	Strongly disagree
S1	My health at present depends on apixaban					
S2	Having to take apixaban worries me					
S3	My life would be impossible without apixaban					
S4	Without apixaban I would be very ill					
S5	I sometimes worry about the long term effects of apixaban					
S6	The apixaban is a mystery to me					
S7	My health in the future depends on apixaban					
S8	The apixaban disrupts my life					
S9	I sometimes worry about becoming too dependent on apixaban					
S10	Apixaban protects me from becoming worse					

Section 2:

Questions relating to your condition for which you are prescribed apixaban

Listed below are a number of symptoms that you may or may not have experienced since your condition for which you have been prescribed apixaban. **Please indicate by circling Yes or No**, whether you have experienced any of these symptoms since your condition **AND** whether you believe that these symptoms are related to your condition. *(Please ensure that you complete **BOTH** columns)*

	**I have experienced this *symptom since my condition***	**This symptom is *related to my condition***
Pain	Yes No	Yes No
Sore Throat	Yes No	Yes No
Nausea	Yes No	Yes No
Breathlessness	Yes No	Yes No
Weight Loss	Yes No	Yes No
Fatigue	Yes No	Yes No
Stiff Joints	Yes No	Yes No
Sore Eyes	Yes No	Yes No
Wheeziness	Yes No	Yes No
Headaches	Yes No	Yes No
Upset Stomach	Yes No	Yes No
Sleep Difficulties	Yes No	Yes No
Dizziness	Yes No	Yes No
Loss of Strength	Yes No	Yes No

Section 2 continued:

We are interested in your own personal views of how you now see your current condition for which you are prescribed apixaban.

Please indicate how much you agree or disagree with the following statements about your illness by ticking the appropriate box.

		Strongly agree	Agree	Uncertain	Disagree	Strongly disagree
IP1	My condition will last a short time					
IP2	My condition is likely to be permanent rather than temporary					
IP3	My condition will last for a long time					
IP4	This condition will pass quickly					
IP5	I expect to have this condition for the rest of my life					
IP6	My condition is a serious condition					
IP7	My condition has major consequences on my life					
IP8	My condition does not have much effect on my life					
IP9	My condition strongly affects the way others see me					
IP10	My condition has serious financial consequences					
IP11	My condition causes difficulties for those who are close to me					
IP12	There is a lot which I can do to control my symptoms					
IP13	What I do can determine whether my condition gets better or worse					
IP14	The course of my condition depends on me					
IP15	Nothing I do will affect my condition					
IP16	I have the power to influence my condition					
IP17	My actions will have no affect on the outcome of my condition					
IP18	My condition will improve in time					

		Strongly agree	Agree	Uncertain	Disagree	Strongly disagree
IP19	There is very little that can be done to improve my condition					
IP20	My treatment (apixaban) will be effective in curing my condition					
IP21	The negative effects of my illness can be prevented (avoided) by my treatment					
IP22	My treatment (apixaban) can control my condition					
IP23	There is nothing which can help my condition					
IP24	The symptoms of my condition are puzzling to me					
IP25	My condition is a mystery to me					
IP26	I don’t understand my condition					
IP27	My condition doesn’t make sense to me					
IP28	I have a clear picture or understanding of my condition					
IP29	The symptoms of my condition change a great deal from day to day					
IP30	My symptoms come and go in cycles					
IP31	My condition is very unpredictable					
IP32	I go through cycles in which my condition gets better and worse					
IP33	I get depressed when I think about my condition					
IP34	When I think about my condition I get upset					
IP35	My condition makes me feel angry					
IP36	My condition does not worry me					
IP37	Having this condition makes me feel anxious					
IP38	My condition makes me feel afraid					

Section 2 continued:

We are interested in what you consider may have been the cause of the condition for which you are prescribed apixaban.

As people are very different, there is no correct answer for these questions. We are most interested in your own views about the factors that caused your condition rather than what others including doctors or family may have suggested to you.

Below is a list of possible causes for your condition. Please indicate how much you agree or disagree that they were causes for you by ticking the appropriate box.

		Strongly agree	Agree	Uncertain	Disagree	Strongly disagree
C1	Stress or worry					
C2	Hereditary – it runs in my family					
C3	A germ or virus					
C4	Diet or eating habits					
C5	Chance or bad luck					
C6	Poor medical care in my past					
C7	Pollution in the environment					
C8	My own behaviour					
C9	My mental attitude e.g. thinking about life negatively					
C10	Family problems caused my condition					
C11	Overwork					
C12	My emotional state e.g. feeling down, lonely, anxious, empty					
C13	Ageing					
C14	Alcohol					

		Strongly agree	Agree	Uncertain	Disagree	Strongly disagree
C15	Smoking					
C16	Accident or injury					
C17	My personality					
C18	Altered immunity					

In the space provided below, please list in rank-order the three most important factors that you believe caused YOUR condition for which you are prescribed apixaban.

You may use any of the items from the boxes above, or you may have additional ideas of your own.

The most important causes for me:

1. ______________________________

2. ______________________________

3. ______________________________

Section 3:

Apixaban therapy might impact on your day to day life. We want to better understand this.

Please answer every question by ticking the box that best describes your views to each statement.

Please remember, there are no “right” answers to the questions, we are simply interested in your views.

During the past 4 weeks…

		Not at all	A little	Moderately	Quite a bit	Extremely
ACTS 1	How much does the possibility of bleeding as a result of apixaban limit you from taking part in vigorous physical activities? (e.g. exercise, sports, dancing, etc)					
ACTS 2	How much does the possibility of bleeding as a result of apixaban limit you from taking part in your usual activities? (e.g. work, shopping, housework, etc)					
ACTS 3	How bothered are you by the possibility of bruising as a result of apixaban?					
ACTS 4	How bothered are you by having to avoid other medicines (e.g. aspirin) as a result of apixaban?					
ACTS 5	How much does apixaban limit your diet? (e.g. food or drink, including alcohol)					
ACTS 6	How much of a hassle (inconvenience) are the daily aspects of apixaban? (e.g. remembering to take your medicine at a certain time, taking the correct dose of your medicine, following a diet, limiting alcohol, etc)					
ACTS 7	How much of a hassle (inconvenience) are the occasional aspects of apixaban? (e.g. the need for blood tests, going to or contacting the clinic/doctor, making arrangements for treatment while travelling, etc)					

Section 3 continued:

Now we want to ask you about daily and occasional aspects of your anticoagulation therapy during the past 4 weeks

		Not at all	A little	Moderately	Quite a bit	Extremely
ACTS8	How difficult is it to follow your anti-clot apixaban?					
ACTS9	How time-consuming is your apixaban?					
ACTS10	How much do you worry about apixaban?					
ACTS11	How frustrating is apixaban?					
ACTS12	How much of a burden is apixaban?					
ACTS13	Overall, how much of a negative impact has apixaban had on your life?					
ACTS14	How confident are you that apixaban will protect your health? (e.g. prevent blood clots, stroke, heart attack, DVT, embolism)					
ACTS15	How reassured do you feel because of apixaban?					
ACTS16	How satisfied are you with apixaban?					
ACTS17	Overall, how much of a positive impact has apixaban treatment had on your life?					

This is the end of the questionnaire

THANK YOU VERY MUCH FOR COMPLETING THIS QUESTIONNAIRE
