# Supplementary material for: Anticoagulated patient’s perception of their illness, their beliefs about the anticoagulant therapy prescribed and the relationship with adherence: impact of novel oral anticoagulant therapy – study protocol for The Switching Study: a prospective cohort study
Source: BMC Hematol. 2016 Aug 23;16(1):22. doi: 10.1186/s12878-016-0061-9 (PMC4995624; doi:10.1186/s12878-016-0061-9)
Supplement: Additional file 4: — Questionnaire packs administered to patients prescribed rivaroxaban*. (PDF 277 kb) [file 12878_2016_61_MOESM4_ESM.pdf]

# Your views on the rivaroxaban therapy prescribed

You have been sent this questionnaire, as you are currently prescribed rivaroxaban therapy. Currently, very little information exists about patient's views on having to take rivaroxaban.

As part of a research study we are conducting within the anticoagulant clinic at King's College hospital, we want to explore your views about rivaroxaban, and would like you to complete this questionnaire.

The questionnaire comprises of three main sections. At the beginning of each section, a short explanation will precede the questions and provide specific instructions on how to complete.

Please answer every question. It will take approximately 20 minutes to complete.

**Your answers will be completely anonymous and will be kept confidential.**

There are no "right" answers to the questions – we are simply interested in your views.

## Thank you for taking the time to complete this questionnaire

**Section 1:**

**Questions relating to medicine use in general**

This section explores your views and concerns (if any) about taking medicines in general.

Please answer every question by ticking the box that best describes your views to each statement.

Please remember, there are no “right” answers to the questions, we are simply interested in your views.

| | | Strongly agree | Agree | Uncertain | Disagree | Strongly disagree |
|---|---|---|---|---|---|---|
| G1 | Doctors use too many medicines | | | | | |
| G2 | Patients who take medicines should stop their treatment for a while every now and again | | | | | |
| G3 | Most medicines are addictive | | | | | |
| G4 | Natural remedies are safer than medicines | | | | | |
| G5 | Medicines do more harm than good | | | | | |
| G6 | All medicines are poisons | | | | | |
| G7 | Doctors place too much trust on medicines | | | | | |
| G8 | If doctors had more time with patients they would prescribe fewer medicines | | | | | |

**Section 1 continued:**

**Specific questions relating to rivaroxaban**

The following questions explore your views and concerns (if any) around taking rivaroxaban specifically.

Please answer every question by ticking the box that best describes your views to each statement.

Please remember, there are no “right” answers to the questions, we are simply interested in your views.

| | | Strongly agree | Agree | Uncertain | Disagree | Strongly disagree |
|---|---|---|---|---|---|---|
| S1 | My health at present depends on rivaroxaban | | | | | |
| S2 | Having to take rivaroxaban worries me | | | | | |
| S3 | My life would be impossible without rivaroxaban | | | | | |
| S4 | Without rivaroxaban I would be very ill | | | | | |
| S5 | I sometimes worry about the long term effects of rivaroxaban | | | | | |
| S6 | The rivaroxaban is a mystery to me | | | | | |
| S7 | My health in the future depends on rivaroxaban | | | | | |
| S8 | The rivaroxaban disrupts my life | | | | | |
| S9 | I sometimes worry about becoming too dependent on rivaroxaban | | | | | |
| S10 | Rivaroxaban protects me from becoming worse | | | | | |

**Section 2:**

## Questions relating to your condition for which you are prescribed rivaroxaban

Listed below are a number of symptoms that you may or may not have experienced since your condition for which you have been prescribed rivaroxaban. **Please indicate by circling Yes or No**, whether you have experienced any of these symptoms since your condition **AND** whether you believe that these symptoms are related to your condition. *(Please ensure that you complete **BOTH** columns)*

| | **I have experienced this *symptom since my condition*** | **This symptom is *related to my condition*** |
|---|---|---|
| Pain | Yes No | Yes No |
| Sore Throat | Yes No | Yes No |
| Nausea | Yes No | Yes No |
| Breathlessness | Yes No | Yes No |
| Weight Loss | Yes No | Yes No |
| Fatigue | Yes No | Yes No |
| Stiff Joints | Yes No | Yes No |
| Sore Eyes | Yes No | Yes No |
| Wheeziness | Yes No | Yes No |
| Headaches | Yes No | Yes No |
| Upset Stomach | Yes No | Yes No |
| Sleep Difficulties | Yes No | Yes No |
| Dizziness | Yes No | Yes No |
| Loss of Strength | Yes No | Yes No |

**Section 2 continued:**

We are interested in your own personal views of how you now see your current condition for which you are prescribed rivaroxaban.
Please indicate how much you agree or disagree with the following statements about your illness by ticking the appropriate box.

| | | Strongly agree | Agree | Uncertain | Disagree | Strongly disagree |
|---|---|---|---|---|---|---|
| IP1 | My condition will last a short time | | | | | |
| IP2 | My condition is likely to be permanent rather than temporary | | | | | |
| IP3 | My condition will last for a long time | | | | | |
| IP4 | This condition will pass quickly | | | | | |
| IP5 | I expect to have this condition for the rest of my life | | | | | |
| IP6 | My condition is a serious condition | | | | | |
| IP7 | My condition has major consequences on my life | | | | | |
| IP8 | My condition does not have much effect on my life | | | | | |
| IP9 | My condition strongly affects the way others see me | | | | | |
| IP10 | My condition has serious financial consequences | | | | | |
| IP11 | My condition causes difficulties for those who are close to me | | | | | |
| IP12 | There is a lot which I can do to control my symptoms | | | | | |
| IP13 | What I do can determine whether my condition gets better or worse | | | | | |
| IP14 | The course of my condition depends on me | | | | | |
| IP15 | Nothing I do will affect my condition | | | | | |
| IP16 | I have the power to influence my condition | | | | | |
| IP17 | My actions will have no affect on the outcome of my condition | | | | | |
| IP18 | My condition will improve in time | | | | | |

| | | Strongly agree | Agree | Uncertain | Disagree | Strongly disagree |
|---|---|---|---|---|---|---|
| IP19 | There is very little that can be done to improve my condition | | | | | |
| IP20 | My treatment (rivaroxaban) will be effective in curing my condition | | | | | |
| IP21 | The negative effects of my illness can be prevented (avoided) by my treatment | | | | | |
| IP22 | My treatment (rivaroxaban) can control my condition | | | | | |
| IP23 | There is nothing which can help my condition | | | | | |
| IP24 | The symptoms of my condition are puzzling to me | | | | | |
| IP25 | My condition is a mystery to me | | | | | |
| IP26 | I don’t understand my condition | | | | | |
| IP27 | My condition doesn’t make sense to me | | | | | |
| IP28 | I have a clear picture or understanding of my condition | | | | | |
| IP29 | The symptoms of my condition change a great deal from day to day | | | | | |
| IP30 | My symptoms come and go in cycles | | | | | |
| IP31 | My condition is very unpredictable | | | | | |
| IP32 | I go through cycles in which my condition gets better and worse | | | | | |
| IP33 | I get depressed when I think about my condition | | | | | |
| IP34 | When I think about my condition I get upset | | | | | |
| IP35 | My condition makes me feel angry | | | | | |
| IP36 | My condition does not worry me | | | | | |
| IP37 | Having this condition makes me feel anxious | | | | | |
| IP38 | My condition makes me feel afraid | | | | | |

**Section 2 continued:**

We are interested in what you consider may have been the cause of the condition for which you are prescribed rivaroxaban.

As people are very different, there is no correct answer for these questions. We are most interested in your own views about the factors that caused your condition rather than what others including doctors or family may have suggested to you.

Below is a list of possible causes for your condition. Please indicate how much you agree or disagree that they were causes for you by ticking the appropriate box.

| | | Strongly agree | Agree | Uncertain | Disagree | Strongly disagree |
|---|---|---|---|---|---|---|
| C1 | Stress or worry | | | | | |
| C2 | Hereditary – it runs in my family | | | | | |
| C3 | A germ or virus | | | | | |
| C4 | Diet or eating habits | | | | | |
| C5 | Chance or bad luck | | | | | |
| C6 | Poor medical care in my past | | | | | |
| C7 | Pollution in the environment | | | | | |
| C8 | My own behaviour | | | | | |
| C9 | My mental attitude e.g. thinking about life negatively | | | | | |
| C10 | Family problems caused my condition | | | | | |
| C11 | Overwork | | | | | |
| C12 | My emotional state e.g. feeling down, lonely, anxious, empty | | | | | |
| C13 | Ageing | | | | | |
| C14 | Alcohol | | | | | |

| | | Strongly agree | Agree | Uncertain | Disagree | Strongly disagree |
|---|---|---|---|---|---|---|
| C15 | Smoking | | | | | |
| C16 | Accident or injury | | | | | |
| C17 | My personality | | | | | |
| C18 | Altered immunity | | | | | |

In the space provided below, please list in rank-order the three most important factors that you believe caused YOUR condition for which you are prescribed rivaroxaban.

You may use any of the items from the boxes above, or you may have additional ideas of your own.

The most important causes for me:

1. ________________________________________

2. ________________________________________

3. ________________________________________

## Section 3:

Rivaroxaban therapy might impact on your day to day life. We want to better understand this.

Please answer every question by ticking the box that best describes your views to each statement.

Please remember, there are no “right” answers to the questions, we are simply interested in your views.

**During the past 4 weeks…**

| | | Not at all | A little | Moderately | Quite a bit | Extremely |
|---|---|---|---|---|---|---|
| ACTS 1 | How much does the possibility of <u>bleeding</u> as a result of rivaroxaban limit you from taking part in <u>vigorous physical activities</u>? (e.g. exercise, sports, dancing, etc) | | | | | |
| ACTS 2 | How much does the possibility of bleeding as a result of rivaroxaban limit you from taking part in your usual activities? (e.g. work, shopping, housework, etc) | | | | | |
| ACTS 3 | How bothered are you by the possibility of <u>bruising</u> as a result of rivaroxaban? | | | | | |
| ACTS 4 | How bothered are you by having to <u>avoid other medicines</u> (e.g. aspirin) as a result of rivaroxaban? | | | | | |
| ACTS 5 | How much does rivaroxaban <u>limit your diet</u>? (e.g. food or drink, including alcohol) | | | | | |
| ACTS 6 | How much of a hassle (inconvenience) are the <u>daily</u> aspects of rivaroxaban? (e.g. remembering to take your medicine at a certain time, taking the correct dose of your medicine, following a diet, limiting alcohol, etc) | | | | | |
| ACTS 7 | How much of a hassle (inconvenience) are the <u>occasional</u> aspects of rivaroxaban? (e.g. the need for blood tests, going to or contacting the clinic/doctor, making arrangements for treatment while travelling, etc) | | | | | |

**Section 3 continued:**

Now we want to ask you about daily and occasional aspects of your anticoagulation therapy during the past 4 weeks

| | | Not at all | A little | Moderately | Quite a bit | Extremely |
|---|---|---|---|---|---|---|
| ACTS8 | How difficult is it to follow your anti-clot rivaroxaban? | | | | | |
| ACTS9 | How time-consuming is your rivaroxaban? | | | | | |
| ACTS10 | How much do you worry about rivaroxaban? | | | | | |
| ACTS11 | How frustrating is rivaroxaban? | | | | | |
| ACTS12 | How much of a burden is rivaroxaban? | | | | | |
| ACTS13 | Overall, how much of a negative impact has rivaroxaban had on your life? | | | | | |
| ACTS14 | How confident are you that rivaroxaban will protect your health? (e.g. prevent blood clots, stroke, heart attack, DVT, embolism) | | | | | |
| ACTS15 | How reassured do you feel because of rivaroxaban? | | | | | |
| ACTS16 | How satisfied are you with rivaroxaban? | | | | | |
| ACTS17 | Overall, how much of a positive impact has rivaroxaban treatment had on your life? | | | | | |

## This is the end of the questionnaire

## THANK YOU VERY MUCH FOR COMPLETING THIS QUESTIONNAIRE
